# Medical information systems: A foundation for healthcare technologies in developing countries

**DOI:** 10.1186/1475-925X-7-18

**Published:** 2008-06-11

**Authors:** Gari D Clifford, Joaquin A Blaya, Rachel Hall-Clifford, Hamish SF Fraser

**Affiliations:** 1 Massachusetts Institute of Technology, Cambridge, MA, USA; 2 Harvard-MIT Division of Health Sciences & Technology, Cambridge, MA, USA; 3 Partners In Health, Boston, MA, USA; 4 Department of Anthropology, Boston University, Boston, MA, USA; 5 Division of Social Medicine and Health Inequalities, Brigham and Women's Hospital, Harvard Medical School, Boston, USA

## Background

Economic disadvantages in developing countries have resulted in health care per capita spending that is almost two orders of magnitude lower than in developed countries [1]. In addition, tertiary-care hospitals in developing countries typically consume a large proportion of overall health care spending, and less than a quarter of government spending is devoted to public health measures and clinical care in primary care settings [2,3]. Community-based care has the capacity for further reaching impact and has been shown to be effective in treatment and monitoring of HIV (Human Immunodeficiency Virus), tuberculosis (TB), and maternal health in resource-poor settings [4-7]. Reliance on community-based care is likely to become even more important as large-scale, chronic disease management is required for HIV and tuberculosis care in settings where acute care or no care at all, is the norm.

In most developing countries, aside from the wealthiest urban areas, the health infrastructure is currently ill-equipped to meet this increasing demand. Although various technologies have been proposed as elements in the solution of this crisis, it is still unclear which technologies have the highest on-the-ground impact and to which settings they are best suited. More extensive data collection concerning medical needs is required to enable the accurate assessment of the effectiveness of interventions and current health care practices.

In 2004, global health spending reached a total of US$ 4.1 trillion. Ninety percent of this total was spent by the 30 wealthy countries of the Organization for Economic Co-operation and Development (OECD), which make up 20% of the world's population [8]. On average, OECD countries spent more than 11% of their gross domestic product on health, while the countries of the World Health Organization's (WHO) African and South-East Asia regions spent 4.7% [8]. In absolute terms, low-income countries spent US$ 32 per capita on health care in 2004, and high-income countries spent US$ 3,724 per capita. Low-income countries health expenditures fall far short of the US$ 60 per capita that the WHO posits is necessary for an adequately functioning health system [9]. Developing countries, like developed countries, face difficult decisions in distributing limited health-care resources. However, this large health care funding gap makes it even more important that low-income countries have optimal resource distribution.

Poverty itself is one of the principal causes of illness in developing countries, and disease in some low-income regions is a significant barrier to economic growth. Poor health causes a spiral of loss of income and is an inhibitor to education [10], which is itself a barrier to obtaining good health (and making good health decisions). Research commissioned by the WHO found that the economic impact of ill health on individuals and societies is far greater than previously estimated [11]. However, they also go on to state that providing basic health care to the world's poor is both technically feasible and cost effective, potentially saving million lives annually and fuelling development by generating hundreds of billions of dollars in new economic activity every year.

As Saxenian points out tertiary-care hospitals in developing countries, alone may consume 30% to 50% percent of overstretched health budgets [2] (although they generally provide the most specialized and sophisticated services and most clinical research, education, and training). Only a quarter of government spending, and often less, is devoted to cost-effective public health measures and to clinical care that is delivered in local health centers and other community settings. This misallocation means that large subgroups in the population, particularly the rural poor, have extremely limited access to health services. Limited government money means that some primary care level treatments are free but more extensive treatment/care can be very costly and cause households to fall into poverty [9]. The World Bank has stressed the value of the primary health care interventions that are commonly included in programs to reduce childhood malnutrition and mortality, chiefly from infectious diseases. However, several of these highly cost-effective interventions have largely been neglected, including: chemotherapy against tuberculosis; integrated prenatal and delivery care; mass programs to de-worm children; and provision of condoms along with information and education to combat AIDS [9].

A recent report by the WHO projects that, over the next twenty years, HIV/AIDS will account for the greatest burden of disease world-wide, followed by depression and ischemic heart disease. Smoking-related illnesses and HIV/AIDS will be the leading causes of death [12]. However, Mathers also points out that this is based upon the assumption that future mortality and risk factor trends in poor countries will have the same relationship to economic and social development as has occurred in the higher income countries over the last 50 years. If this assumption is wrong, then predictions may be worse. Therefore, in order to best allocate resources, tracking of health care problems and the evaluation of prevention and treatment programs (particularly where HIV/AIDS is concerned) as a function of local economics and social attitudes is essential. Information technology has been proposed as an efficient method for improving the effectiveness and efficiency of health care [13], and has been shown to be particularly useful in the context of outcome improvement, cost reduction [14] and disease intervention [15-20]. This article therefore concentrates, not on what health care programs and devices are likely to be useful, but on how information technology can be employed to improve our understanding of what technologies and practices are needed, while addressing specific problems, such as information loss (and errors), long latencies in delivery, and the cost of health care provision.

## Tracking Health Care, Databases and Information Systems

Information technology and electronic medical records (EMRs) have been shown to provide significant benefits in developed countries. Studies have shown that it can improve patient outcomes in the management of renal disease [14,21]. In another recent study of almost a million patients in the Colorado and Northwest regions of the Kaiser Permanente health care system, two years after electronic health records were fully implemented, age adjusted rates of office visits were shown to be 9% lower in both regions [22]. Age adjusted primary care visits were shown to drop by 11% in both regions and specialty care visits decreased by 5% in Colorado and 6% in the Northwest. All decreases were significant (P < 0.0001). Wang *et al*. [23] have estimated that the net benefit from using an electronic medical record for a 5-year period was $86,400 US per provider. Benefits accrue primarily from savings in drug expenditures, improved utilization of radiology studies, better capture of charges, and decreased billing errors. A recent long term study of the US Veterans Health Administration (VHA) has demonstrated that EMRs improve efficiency by an estimated 6% per year, and that the only a small number of unnecessary tests or admissions resulted from the usage of their EMR [24].

Although large differences exist between infrastructure and resources for health care in developing countries [25-27], it is possible that EMRs are able to provide similar impacts on health care in developing countries. In fact, given the poor state of medical record keeping in many developing regions, EMRs may even lead to much larger impacts on outcomes, health care efficiency and treatment delivery in developing countries [28-31].

In 2005, Eiseman and Fossum pointed out that available health resource data for developing countries is currently a "patchwork of information at different levels of aggregation and resolution and of varying quality and timeliness that falls short in meeting the needs of the many diverse objectives and organizations that require such data" [32]. Furthermore, "many current data collections rely on labor-intensive collection techniques that require extensive planning and the skills of specially trained teams, which can prove burdensome to those providing the data and may be detrimental to the data's accuracy and timeliness". Eiseman and Fossum and others go on to point out that existing data collections are insufficiently comprehensive, sometimes inaccurate, and often out of date by the time the data can be acted upon [32] ([33]. Without such data, none of the parties trying to address the health problems of developing countries has the required empirical knowledge to inform policy decisions about health resource mobilization and allocation, strategic planning, priority setting, monitoring and evaluation, advocacy, and general policymaking [34-36].

Eiseman and Fossum propose that any global health resource tracking system would contain valid, detailed data (who, what, where, how much) on all health resources (cash and in-kind) provided in previous, current and the next fiscal years to all developing countries by all public and private entities. Furthermore, this should be provided in (almost) real time, without double-counting any resources. Such a system should also have the following ideal properties:

1. Impose on any public or private entity no more than a minimal burden in terms of its provision of the information needed to populate the system.

2. Readily harmonize with and connect to the existing data systems of receiving countries and all donor entities.

3. Be easily accessible via the Web and flexibly searchable by every data element in a variety of languages.

4. Enjoy broad ownership, official buy-in, and use, with long-term support from a diversified funding base.

Eiseman and Fossum point out that this would require that practical data systems already exist in a meaningful way and that they are easily accessible to the relevant users. The reality is that most countries do not use digital health records, and even those who do, often have an extremely limited ability to facilitate searches and exchange data with other systems [37,38]. Furthermore, there is no clear consensus on how the data should be collected, and in what format should the data be stored. These are two key issues in the development of a useful medical health record.

Essentially, there are two possible approaches to the storage of data in an EMR. The first prescribes a top-down national (or international) schema for the medical data, such as the GEHR/openEHR standard, the CEN EN 13606 EHRcom standard, and the HL7 standard. (See Sanroma *et al*. [39] for a good overview of these standards.) The major disadvantages of these approaches are that they are difficult to implement for small projects and are not always suited to primary care-level information collection. An alternative approach is to employ a system that is built from the bottom-up, such as OpenMRS [40-45]. These approaches lead to a streamlined system that provides only for the needs of the project, with little overhead. However, the system is also standardized (for integration with other software, and databases) and extensible so that other data can easily be added to the system.

In both approaches, the EMR should be built with open-source software. This has several advantages over closed proprietary systems. Firstly, the system is more 'future-proof', being able to withstand the changes in libraries, operating systems and hardware. This avoids the problems of having to reverse-engineer data structures and recode interfaces. Furthermore, software can be written in a cross-platform manner, providing maximum choice and flexibility for users. Secondly, open-source software is license free and allows everyone to benefit from any developments made by others, minimizing the costs to everyone involved. Vital funds can then be spent on the support and augmentation of the code base. Use of open-source software can also lead to an increase in competition and allow developing countries to support their own software and applications and the development of related businesses. Examples of such competition stimulation can be seen in the adoption of Linux and Apache by the Apple Corporation. Furthermore, open-source licensing can allow small and medium-sized companies to build a business around the support of medical databases. It should be noted that open-source does not always mean that software is always supplied at no-cost to the user, and such software can be linked to proprietary libraries if the source-code base is distributed under an appropriate license, such as the modified BSD or LGPL licenses. Thirdly, it is generally easier to detect and fix bugs in open-source software, and compliance with standards is more easily enforced. In particular, standards concerning security and protected health information are more easily audited when a system is open.

Since electronic data flow must involve hardware at some point, hardware communication issues must also be considered. The problems of integrating hardware with proprietary interfaces and back-end databases are well-known in the developed world, and these issues do not benefit either the patient or the health care system. That is not to say that private enterprise's role in healthcare in developing countries is unimportant (and we leave this involved discussion for another time), but the foundations of device communication should be sufficiently open in order to maximize the usefulness of any medical record system.

In any EMR, it is also advantageous to include standardized medical languages (such as the Unified Medical Language System; UMLS), which have multiple-language translations, enable multi-lingual versions of the electronic health record and help aggregate data across regions and nations. Errors due to regional differences in the names of drugs, or colloquial terminology for procedures can lead missed opportunities to treat or even to serious medical errors.

## Successful technology implementation requires multi-factorial approach

Unlike in developed countries where technology implementation can be focused and rely on existing infrastructure, in developing countries a multi-factorial approach is necessary if technology is going to be implemented and maintained successfully. Among some of the factors that must be taken into account are, corruption, inequalities within the country, imposition of sub-optimal policies or technologies by authorities, and the lack of or incorrect information. Corruption plagues health systems in all countries. In developing countries, one common form is the requisite informal payments to underpaid health staff which creates a significant barrier to care [9]. A 2003 study of the government health system in Albania found that treatment was withheld in the absence of an informal payment and that patients included the estimated costs of informal payments in their decisions to seek care [46,47]. A conceivable offshoot of this established corruption in light of improved health care technology would be the misuse of technology resources or the increase of existing informal payments for their use. In establishing technology-focused health programs in developing countries, monitoring of users as well as data collection and entry must be a significant concern, beginning in the planning stages of program implementation.

In conceptualizing technology-based improvements to health care in the developing world, it is essential to bear in mind the disparities of access and quality of care that currently exist within the health system in any given country and how the planned improvements may exacerbate those inequities. If technological improvements are centered on urban areas, they will likely not impact the health status of the rural poor and, thus, may only marginally benefit the country's overall health indicators [48]. On an implementation level, the strata of the health care hierarchy at which technological advancements will be made are important to consider on the front-end, as this will impact the needed hardware and user-interface. Further, in targeting the end-user population early in the development phases of a technology-based intervention, the health care staff, be they doctors, nurses, or community health workers, can be made a central part of the planning and implementation teams. If the end-users do not perceive a need or value to a new piece of technology, the overall success of implementing that technology will likely be low.

As Malkin [25-27] points out, problems such as rising costs of medical equipment, embedded service contracts, lack of spare parts, lack of required consumables, lack of reliable power and water, lack of public infrastructure such as roads, and lack of technical expertise, plague health care technology in the developing world. While poor infrastructure, such as the telecommunications and electricity grid, should not be seen as justification for relegating improvements in health care technology to the future, the realities of existing capacities must be taken into account. For example, many developing countries have far more reliable wireless than traditional telecommunications systems [49], and technological advancements should focus on those existing strengths. Information and communications technology (ICT) is one area in which developing countries have made significant advancements, and it has been touted as a cost-effective mechanism for delivering health care information in developing countries [50]. In particular, ICT can be leveraged to address the dearth of trained personnel, by both interpreting medical data and facilitating training. With a continually growing rate of over 80% of the world's population living in range of a cellphone tower, telemedicine applications for automated or remote analysis (such as X-ray reading [51]) are becoming increasingly attractive.

In order to illustrate the above points, examples of successful applications of ICT to health care for under-served populations in Peru and Haiti are described in the following section.

## The PIH Projects in Peru and Haiti: Health ICT examples in middle and low income countries

In 1996 Partners In Health (PIH), with their Peruvian sister organization, Socios En Salud (SES) and the Peruvian Ministry of Health, started a community-based treatment program for drug-resistant tuberculosis in the slums of Lima, Peru. Multi-drug resistant tuberculosis (MDR-TB) is defined as TB resistant to isoniazid and rifampin, the two most efficacious anti-tuberculous drugs. At that time the few models for treatment of MDR-TB were costly and were centered around referral hospitals. Reported rates of success in middle-income countries and regions ranged from less than 60% in Indonesia and Taiwan [52,53] to just over 80% in Hong Kong, Korea, and Turkey [54-57]. PIH and SES created a community-based project to treat MDR-TB in a resource-poor setting. This new project termed "DOTS-Plus" project built on top of the well-established Peruvian Directly Observed Therapy-Short Course (DOTS) program and treated patients with long-standing disease due to highly resistant strains of TB.

Reported cure rates in this community-based, ambulatory program were as high as any reported in a hospital setting to date [7]. Unlike other cohorts, which had high default rates, all patients in the PIH/SES cohort received directly observed therapy. Adverse effects, moreover, were carefully managed to ensure completion of treatment. Mitnick et al. [7] state that in 1997 "mean treatment costs were US$15,681 per patient, these costs were low at that time – approximately 10% of the costs for hospitalized patients [58,59] – but well beyond the reach of most national tuberculosis programs." Since then advocacy work and pooled procurement have made second-line anti-tuberculous drugs available to countries and programs needing them. Through negotiations with the research-based and generic pharmaceutical industries, the cost of drugs for multi-drug-resistant TB was reduced by up to 98% [60]. By establishing a long-term collaboration and moving treatment into the community, PIH was able to provide high-quality care, lower costs, reduce the risk of nosocomial spread of MDR-TB [61-64], and provide additional, individualized services that patients in low-resource areas may need. Further, this community-based network can be strengthened to provide primary care [65] and be a source of data for further interventions.

The Partners In Health Electronic Medical Record (PIH-EMR) [17], implemented in 2001, was developed to support the two-year MDR-TB treatment regimen for the cohort described above. The PIH-EMR is an example of a web-based EMR based on open-source technology and backed by an Oracle database. The system is viewable in both English and Spanish and currently has over 29,000 patients, 7,600 of which have received treatment. The PIH-EMR includes a clinical record with initial history, physical examination, laboratory results and medications on all patients receiving individualized treatment for MDR-TB. The custom medication order entry system provides advice on potential problems and feedback to the clinical personnel. There is an extensive suite of web-based analysis tools for reporting and outcome monitoring [17]. Analysis tools are used to assess drug requirements based on the medications prescribed and perform operation research. It is also linked to a pharmacy inventory and dispensing system. Evaluations of modules of this system have shown that the medication order entry system produced significantly fewer errors than the previous paper and spreadsheet approach [15]. Drug usage prediction tools have been shown to match the usage data in the pharmacy to within 3% [16] and are used routinely is drug ordering. Further modules have been added to the PIH-EMR to collect and communicate TB laboratory data. A personal digital assistant (PDA)-based system to collect TB lab data from laboratories and health centers without internet was shown to reduce processing delays from 30 to 8 days, reduce errors by 60%, and to be preferred by users [66].

The PIH-EMR has recently been adopted by the Peruvian National Tuberculosis Program for its drug-resistant TB treatment program, and there are plans to expand its use to the entire TB program in Peru. The PIH-EMR is also used to create monthly reports for the Global Fund and the Health Ministry. This experience demonstrates that these types of systems are feasible to implement in resource-poor settings. Another web-based module termed e-Chasqui has been designed and implemented to improve the timeliness and quality of laboratory data [28]. In Peru, the e-Chasqui system has been deployed in the national TB laboratory, two regional laboratories, and 24 pilot health centers. Since its full implementation in March 2006, over 70,000 TB laboratory tests have been entered into the system with over 99% of them viewed online by the health centers. In total, e-Chasqui serves a network of institutions providing medical care for over 3.1 million people at a cost of approximately US$0.53 per sample, the annual total cost is equivalent to 1% of the National Peruvian TB program's 2006 budget.

Since 1999, PIH has run a community-based HIV treatment program in Haiti with its sister organization, Zanmi Lasante, expanding to nine public health clinics in an area with virtually no roads, electricity or telephone service. In these clinics, 'Directly Observed Therapy with Highly Active Antiretroviral Therapy' (DOT-HAART) for HIV is modeled on successful tuberculosis control efforts like the one described previously. Each HIV patient has a community-health worker who observes ingestion of pills, responds to patient and family concerns, and offers moral support. Social support – including assistance with children's school fees – is included in services offered. Monthly meetings, in which patients discuss their illness and other concerns, are notable for high attendance [4,67]. In 2006 over 8,000 HIV-positive persons, 2,300 of whom are on antiretroviral therapy (ART), are now followed [68]. Adherence to HAART was very high, and clinical outcomes were excellent: all patients responded with weight gain and improved functional capacity, and fewer than 5% required medication changes due to side effects [69]. As elsewhere, patients receiving HAART are far less likely to require admission to hospital than are patients with untreated HIV disease [70].

The HIV-EMR, an open source web-based system [71], was based on the PIH-EMR. Satellite-based internet access at each site provides access to the system; however, due to the inconsistent power and internet available, an additional offline client for data entry and review was implemented [72]. The HIV-EMR system has been implemented in all sites and currently has over 12,000 patients; 3,051 of which are receiving ART. The system records clinical data including history, physical examination, social circumstances and treatment prescribed. Decision support tools provide allergy and drug interaction warnings and generate warning emails about low CD4 counts. (The lower your CD4 count, the greater the chances of potentially fatal infections.) Staff also keep paper records, but they can use the EMR to check for up to date lab results and drug regimen data and monitor patients' follow-up status. A suite of reporting tools allow staff to create key reports, such as for the U.S. President's Emergency Plan for AIDS Relief (PEPFAR), and automatically generate the reports monthly. Data quality is backed by a monthly checklist of patients and their drug regimens and treatment status that is filled out by the pharmacy and nursing staff and used to update and cross-check the EMR. There is also a full pharmacy inventory system and tools for drug regimen analysis. The inventory system allows pharmacy staff at all clinics to enter stock levels and request drugs and track shipments. This system is used to track 450 products supporting care for 1.78 million patient visits annually. Over the last year drug stockouts have fallen from 2.6% to 1.1% and 97% of stock requests delivered were shipped within 1 day [73]. EMR systems have been shown to provide a better one-year estimate for medication ordering and therefore reduce costs in having stock-outs or more expensive, local emergency purchases compared to the current method of ordering based on the last year's estimates [74,75].

## Summary

Among the significant barriers to the provision of health care in developing countries, detailed information concerning disease incidence, health practices and available resources (such as drugs for treatment) are some of the most important. Without detailed information concerning the response to health programs, it is impossible to evaluate the efficacy of a particular program and, hence, effectively allocate funding and resources. Although paper-based systems can provide a partial solution, information transmission is slow and prone to errors. Furthermore, aggregation of data is challenging as patient numbers rise into the hundreds [19], and near impossible with thousands of patients. It is also difficult to impose consistent reporting indicators.

The systems described above illustrate the advantages of implementing healthcare technologies within larger collaborations that improve the overall public health infrastructure. One key aspect of the technologies employed in these projects is the use of open standards and open-source development in a collaborative environment.

The cases described in this article also demonstrate the need for community data collection, and feasibility of using ICT to enable data collection, and improve information flow in developing countries. Without such approaches, interventions may exacerbate inequalities within countries with weak infrastructure and ingrained social disparities. However, these systems will only work well with carefully designed forms and interfaces, and excellent data management. Furthermore, EMRs can provide a foundational technology that allows for the adoption and evaluation of other health care technologies, such as drug ordering, medical devices, and longitudinal patient follow-ups. Moreover, the projects described above illustrate that the creation of long-term relationships to build infrastructure and solving systemic problems to provide health care can be beneficial to both the patients and the projects involved.

## Abbreviations

AIDS: Acquired Immune Deficiency Syndrome; ART: Antiretroviral Therapy; BSD: Berkeley Software Distribution; CEN: Comité Européen de Normalisation; CD4: Cluster of Differentiation 4; DOTS: Directly Observed Therapy-Short course; DOT-HAART: Directly Observed Therapy with Highly Active Antiretroviral Therapy; EHR: Electronic Health Record; EMR: Electronic Medical Record; GEHR: Good Electronic Health Record; HIV: Human Immunodeficiency Virus; HL7: Health Level 7; ICT: Information and Communications Technology; ICT4D: Information and Communications Technology for Development; LGPL: Lesser GNU Public License; MDR-TB: Multi-Drug Resistant Tuberculosis; MRS: Medical Record System; OECD: Organization for Economic Co-operation and Development; PDA: Personal Digital Assistant; PEPFAR: President's Emergency Plan for AIDS Relief; PIH: Partners In Health; PIH-EMR: Partners In Health Electronic Medical Record; SES: Socios En Salud; TB: Tuberculosis; UMLS: Unified Medical Language System; VHA: Veterans Health Administration; WHO: World Health Organization.

## Competing interests

The authors declare that they have no competing interests.

## Authors' contributions

GDC drafted the editorial and provided the final editing, RHC contributed material and references concerning anthropology and public health statistics as well as editorial assistance, JAB and HSFF made significant changes and provided material and references concerning PIH research in Haiti and Peru. All authors read and approved the final manuscript.
